# Molecular Subtypes and Personalized Therapy in Metastatic Colorectal Cancer

**DOI:** 10.1007/s11888-016-0312-y

**Published:** 2016-04-18

**Authors:** Donna M. Graham, Vicky M. Coyle, Richard D. Kennedy, Richard H. Wilson

**Affiliations:** Centre for Cancer Research and Cell Biology, Queen’s University Belfast, 97 Lisburn Road, Belfast, BT9 7AE N. Ireland UK

**Keywords:** Colorectal cancer, Biomarker stratification, Targeted therapy, Pathways, Molecular subtypes, Personalized medicine trials, Microsatellite instability, RAS, BRAF, EGFR, c-MET, HER2

## Abstract

Development of colorectal cancer occurs via a number of key pathways, with the clinicopathological features of specific subgroups being driven by underlying molecular changes. Mutations in key genes within the network of signalling pathways have been identified; however, therapeutic strategies to target these aberrations remain limited. As understanding of the biology of colorectal cancer has improved, this has led to a move toward broader genomic testing, collaborative research and innovative, adaptive clinical trial design. Recent developments in therapy include the routine adoption of wider mutational spectrum testing prior to use of targeted therapies and the first promise of effective immunotherapy for colorectal cancer patients. This review details current biomarkers in colorectal cancer for molecular stratification and for treatment allocation purposes, including open and planned precision medicine trials. Advances in our understanding, therapeutic strategy and technology will also be outlined.

## Background

Treatment of metastatic colorectal cancer (CRC) with traditional 5-fluorouracil (5FU)-based chemotherapy has been modified, with addition of oxaliplatin, irinotecan and monoclonal antibodies targeting vascular endothelial growth factor (VEGF) and the epidermal growth factor receptor (EGFR). These modifications have led to improved overall survival for patients to over 40 months from diagnosis [1–3]. However, considerable heterogeneity exists within CRC due to the varied genetic and epigenetic mechanisms involved in differing pathways of colorectal carcinogenesis [4]. Improved understanding of common tumours, for example breast cancer, has resulted in stratification for prognostication and treatment purposes being used for over a decade [5]. In non-small cell lung cancer (NSCLC), single gene “driver” mutations predict for high response rates using molecularly targeted agents [6, 7]. However, attempts to stratify CRC using clinicopathological and molecular features for prognostic and predictive purposes have had limited success, and correlation between subtyping strategies has been poor [8–10]. This review will discuss molecularly defined subtypes of CRC and implications for current and future patient management.

## Colorectal Carcinogenesis

**Fig. 1:**
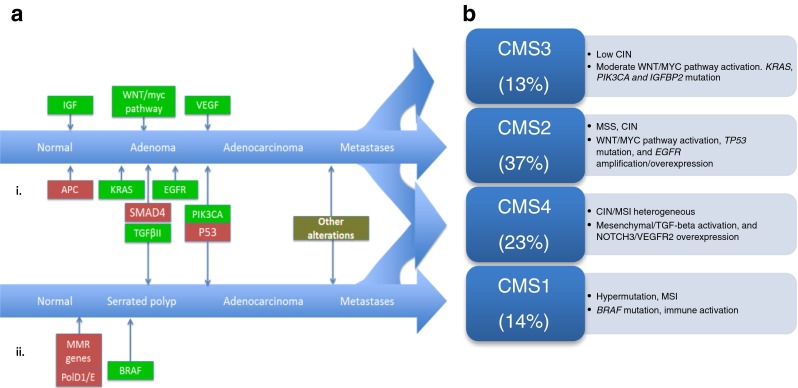
**a** Genetic models of CRC: *i*. Chromosomal Instability; *ii*. Mutator phenotype. Stepwise carcinogenesis occurs due to differing molecular changes in each model. **b** Resulting core genomic subtypes by molecular subtyping. *MMR* mismatch repair, *CIN* chromosomal instability, *MSS* microsatellite stable, *MSI* microsatellite instable

Fearon and Vogelstein proposed a stepwise model for colorectal carcinogenesis highlighting the role of critical tumour suppressor genes and oncogenes in adenoma and subsequent carcinoma development with inactivation of the *adenomatous polyposis coli* (APC) tumour suppressor gene as the initiating event [11]. This is understood to be the predominant mechanism for development of CRC. Subsequently, less frequently occurring tumourigenic pathways have been identified. The processes have been classified as either chromosomal instability (CIN) or mutator phenotype that includes DNA repair defects and aberrant DNA methylation [4]. Characteristic genomic changes are associated with each of these (Fig. 1a).

## Microsatellite Instability

Protective mechanisms exist to facilitate replication of normal cellular DNA. Genes encoding for DNA damage repair (DDR) proteins are commonly mutated in cancer. Dysregulation of this process permits an accumulation of somatic mutations and contributes to genomic instability, now recognised as critical in cancer development and metastasis [12]. As a pathway involved in both hereditary and sporadic forms of CRC, the mutator phenotype with mismatch repair deficiency (dMMR) is of particular interest.

Postreplicative mismatch repair (MMR) maintains genomic stability by eliminating single-base mismatches and insertion-deletion loops of short repeated nucleotide sequences (microsatellites) occurring during DNA replication [13]. Normal interaction of critical MMR proteins comprising MLH1, MSH2, MSH3, MSH6 and PMS2 is required for proofreading and correction of these insertion-deletion loops. Mutation of genes encoding for these MMR proteins or hypermethylation of the *MLH1* promoter causes gene silencing, with dMMR and subsequent microsatellite instability (MSI). Due to lack of recognition and clearance of damaged cells during replication, dMMR status results in an accumulation of mutations within tumour cells compared to those with proficient MMR (pMMR) or microsatellite stability (MSS) that facilitates tumour growth [14].

The MSI subgroup constitutes approximately 15 % of CRC overall. Germline mutation of MMR genes may occur as an autosomal dominant syndrome termed hereditary non-polyposis colorectal cancer (HNPCC), or Lynch syndrome, and contributes to 2–5 % of CRC. *MLH1* is most frequently involved either by mutation in 60 % of inherited cases or epigenetic inactivation in association with the CpG island methylator phenotype (CIMP) [15]. MSI CRCs are more frequently located in the proximal bowel, with poor differentiation, mucinous subtype and dense lymphocytic infiltration, suggestive of a strong antitumoural immune response [16]. Patients with sporadic MSI CRC are older and the tumours are characterised by frequent *BRAF* (V600E) mutation and absence of MLH1 and PMS2 proteins [17]. Loss of MLH1 expression increases with age, resulting in CRC development in the elderly, in contrast to earlier disease presentation in patients with Lynch syndrome [18, 19].

DNA polymerases δ and ε are involved in DNA reconstruction following damage. Germline variants in the exonuclease domain of the DNA polymerases POLE and POLD1 predispose to cancer including CRC, by impairing polymerase proofreading and greatly increasing the rate of base substitution mutations. The resulting tumour phenotype has similarities to that caused by dMMR, including high immunogenicity and favourable prognosis but with mutation burden significantly higher than that found in MSI CRC [20, 21]. These are a rare subtype (0.25–1.5 % of CRC) and no therapeutic strategies currently exist to target these specific genomic aberrations, although such investigation is planned (Table 1 and Table 2).

### Prognostic and Predictive Implications for MSI

MSI status has been evaluated as a prognostic biomarker in CRC, with conferral of prognostic benefit for patients with early MSI CRC [22]. Molecular subtyping strategies have highlighted a lower disease-specific mortality in patients with MSI CRC regardless of the initiating defect [23]. This benefit appears to hold true in the adjuvant setting. However, a subsequent study of patients with stage II and III disease showed no significant difference in overall survival (OS) or progression-free survival (PFS) due to dMMR, but demonstrated statistically poorer OS following administration of 5FU-based chemotherapy (82.4 % compared with 89.5 % for pMMR) [24]. It has been proposed that adjuvant 5FU-based chemotherapy should be avoided in stage II MSI CRC where toxicity may be prevented. Whether the addition of oxaliplatin may abrogate the detrimental effect of 5FU for patients with MSI CRC remains unclear [25–29]. The prognostic benefit of MSI in early stage disease is lost at time of relapse with rapid disease progression and reduced OS [30].Given their possibly poorer response to 5FU-based chemotherapy, use of targeted agents is of great interest in this subgroup.

Poly [ADP-ribose] polymerase-1 (PARP-1) localizes to sites of DNA damage and is involved in repair of these defects. In MLH1*-* and MSH3-deficient CRC cell lines, the combination of PARP inhibition and irinotecan therapy was more effective than in those with functional MLH1 [31, 32]. Consequently, there is interest in use of PARP inhibition for treatment of MSI CRC (Table 1).

**Table 1:** Selected molecular subtypes of colorectal cancer and associated clinical trials utilising targeted agents

Tumour subtype	Investigative strategies	Agents	Phase of trial	Clinicaltrials.gov identifier
Mismatch repair deficient/microsatellite instable	PARP inhibition	Olaparib	II	NCT00912743
	Immune checkpoint inhibition (PD-L1)	Durvalumab (MEDI4736)	II	NCT02227667
	Immune checkpoint inhibition (PD-L1)	Atezolizumab (MPDL3280A)	II	NCT02291289
	Immune checkpoint inhibition (PD-1)	Nivolumab ± Ipilimumab	I/II	NCT02060188
	Immune checkpoint inhibition (PD-1)	Pembrolizumab (MK-3475)	II	NCT02460198
		± standard chemotherapy	III	NCT02563002
*RAS* mutation	Pan-RAF inhibition	BMS-908662 ± Cetuximab	I/II	NCT01086267
	AKT and MEK inhibition	MK-2206 and AZD6244 (Selumetinib)	II	NCT01333475
	HDAC inhibition	4SC-201 (Resminostat) + FOLFIRI	II	NCT01277406
	ERK inhibition	CC-90003	I	NCT02313012
	NOTCH inhibition	RO4929097 + Cetuximab	I	NCT01198535
	MEK and MET inhibition	PD-0325901 + Crizotinib	I	NCT02510001
	Multikinase inhibition	Regorafenib	II	NCT02175654
	MEK and BCL2 inhibition	Trametinib and Navitoclax (ABT-263)	I/II	NCT02079740
	PI3K and MEK inhibition	BKM120 + MEK162	I	NCT01363232
Resistant to EGFR inhibition	RAF inhibition	BMS-908662 ± Cetuximab	I/II	NCT01086267
	NOTCH inhibition	RO4929097 + Cetuximab	I	NCT01198535
*BRAF* mutation	PI3K and MEK inhibition	BKM120 + MEK162	I	NCT01363232
		BEZ235 + MEK162	I	NCT01337765
	BRAF, MEK and EGFR inhibition	Trametanib, Dabrafenib + Panitumumab	II	NCT01750918
	RAF inhibition	BMS-908662 ± cetuximab	I/II	NCT01086267
	BRAF and EGFR inhibition	Irinotecan, Cetuximab + Vemurafenib	II	NCT02164916
	BRAF, PI3K and EGFR inhibition	LGX818, BYL719, and Cetuximab	I/II	NCT01719380
	BRAF, WNT and EGFR inhibition	LGX818, WNT974 and Cetuximab	I/II	NCT02278133
	ERK inhibition	BVD-523	I	NCT02313012
	ERK inhibition	CC-90003	I	NCT02313012
*PI3K* mutation/PTEN depletion	MEK and PI3K/mTOR inhibition	Pimasertib + SAR245409	I	NCT01390818
	AKT and MEK inhibition	MK-2206 and AZD6244 (selumetinib)	II	NCT01333475
HER2 amplification	HER2 dual inhibition	Trastuzumab + Lapatinib	II	EudraCT Number: 2012-002128-33
		Trastuzumab + Pertuzumab	II	
MET amplification	MEK and MET inhibition	PD-0325901 + Crizotinib	I	NCT02510001

Lymphocytic infiltration as a characteristic feature of MSI CRC has prompted further investigation due to the increasing availability of effective immunotherapies. The reasons for this immunogenic phenotype are unclear. However, it is postulated that this may occur due to the creation of tumour-specific neoantigens during accumulation of mutations [33]. Analysis of primary tumour tissue from this patient subset identified high levels of infiltration of activated CD8+ cytotoxic T-cells and activated Th1 cells with interferon-γ (IFNγ) and T-box expressed in T cells (TBET), a Th1 transcription factor. In addition, upregulated expression of the immune checkpoints cytotoxic T lymphocyte-associated antigen 4 (CTLA4), programmed cell-death-1 (PD-1), programmed cell-death ligand-1 (PD-L1), lymphocyte activation gene 3 (LAG3) and indoleamine 2,3-dioxygenase (IDO) was noted in MSI tumours [34•]. The critical role of the immune system in tumour regulation has been widely highlighted [12] and lymphocytic infiltration in CRC is associated with lower rate of relapse and improved prognosis [35]. Current immunotherapeutic strategies incorporate antibodies to directly inhibit the CTLA4 and PD-1 pathways. Use of the PD-1 inhibitor pembrolizumab has been associated with responses in patients with MSI CRC [36••]. Further studies of immune checkpoint inhibitors are ongoing in this patient population (Table 1).

## Individual Somatic Mutations

### RAS Mutation

The mitogen-activated protein kinase (MAPK) pathway comprised of the RAS-RAF-MEK-ERK signalling cascade is deregulated due to somatic gene mutation in >50 % of CRC, with 40 % of these due to activating mutations in *KRAS* [37]. This pathway lies downstream of the epidermal growth factor receptor (EGFR). Activating mutations in this pathway will result in transcription of the gene for transforming growth factor-α (*TGFα*), a ligand of the EGFR. This creates an autocrine signalling loop that contributes to tumoural resistance to the anti-EGFR monoclonal antibodies (mAbs), cetuximab and panitumumab [38]. In combination with chemotherapy, these therapies are the only approved biomarker-driven therapies currently licensed for treatment of CRC and have extended survival up to 41.3 months in patients with tumours wild type (WT) for *KRAS* and *NRAS* [3, 39]. However, despite improved survival for a subset of patients, no benefit is seen for those with *KRAS/NRAS* mutated tumours. Absence of this mutation is not an accurate predictor of response, as only 40–50 % of patients with *KRAS/NRAS* WT disease respond to the anti-EGFR monoclonal mAbs [40]. Binding of amphiregulin (AREG) and epiregulin (EREG) ligands to the EGFR stimulate mitogenesis [41]. In *KRAS* WT CRC, high expression of AREG and EREG messenger RNA (mRNA) is predictive of improved response rate, progression-free survival and overall survival in patients treated with cetuximab [42–44]. High EREG mRNA expression levels may predict for response in patients irrespective of EGFR mutation status [44]. However, these biomarkers are not validated for use in clinical practice.

Initially, mutations conferring resistance to anti-EGFR mAbs were identified in codons 12 and 13 of exon 2 of the *KRAS* gene. However, retrospective analysis of multiple trials demonstrated the presence of further resistance mutations in exons 2, 3 and 4 of *KRAS/NRAS*, resulting in a recommendation to utilise wider *RAS* mutational testing prior to treatment selection and inclusion in the license for panitumumab and cetuximab [45]. The *KRAS* G13D mutation conveys sensitivity to EGFR inhibitors in preclinical models, and retrospective clinical reports suggested possible treatment benefit with cetuximab similar to *KRAS* wild-type CRC. However, the prospective international ICECREAM trial recently confirmed lack of efficacy of cetuximab in this molecular subgroup [46].

Acquired mechanisms of resistance may limit treatment efficacy of EGFR inhibitors. Use of circulating tumour DNA (ctDNA) has identified emergence of *RAS* mutations following cetuximab administration, conferring resistance to EGFR-targeted therapy. Interestingly, introduction of an *MEK* inhibitor in combination with cetuximab resensitised the tumour to anti-EGFR therapy [47].

A number of other primary resistance mechanisms against anti-EGFR mAbs exist, including downstream activation of *BRAF* and *MEK*. In addition, EGFR activation may occur via an alternate signalling pathway within the interlinked RAS-MAPK-PI3K or may be mediated by type 1 insulin-like growth factor receptor (IGF-1R). Reduced affinity of the EGFR ligand or mutations within the gene, resulting in a conformational change to the binding site, may also physically impair the ability of the monoclonal antibody to effectively bind to and inhibit activation of the EGFR [48]. Based upon preclinical research demonstrating HER2 as a resistance pathway to EGFR inhibitors, the HERACLES trial investigates dual inhibition of the HER2 pathway and the efficacy of dual HER2 targeting in patient-derived xenografts [49]. The rate of HER2 positivity was 5.4 % in the first cohort, with a 34 % response rate and 44 % stable disease from trastuzumab and lapatinib in 23 patients. Trials investigating additional targeted agents aimed at reducing resistance to anti-EGFR inhibitors are currently underway (Table 1).

Due to the relative frequency of *RAS* mutation in CRC and its use as a negative predictor for current molecularly stratified treatment, identification of an effective therapy to target this subgroup has potential clinical benefit. Treatment directed at *RAS* mutated CRC has been targeted against downstream *MEK* signalling, but has been less successful than treatment for *KRAS* WT disease. Inhibition of *MEK* signalling may cause upregulation of signalling via the PI3K-AKT-mTOR pathway [50]. *RAS* is also an oncogenic driver of the PI3K-AKT-mTOR pathway, providing rationale for the combination of *RAS* and *MEK* pathway inhibition as a potential therapeutic strategy (Table 1).

### BRAF Mutation

An additional activating mutation implicated in tumourigenesis within the MAPK signalling cascade is *BRAF* V600E mutation. This occurs in 8–10 % of CRC, very rarely occurs in conjunction with *RAS* mutations, and confers resistance to anti-EGFR therapy [51]. Patients with these tumours have a poor prognosis in the metastatic setting with aggressive tumour biology. This mutation is strongly associated with sporadic MSI CRC [30]. Targeted *BRAF* blockade using single-agent tyrosine kinase inhibition was attempted for this disease subtype. However, this approach was unsuccessful, with preclinical data suggesting therapeutic failure was due to aberrant upstream signalling via *MEK* and activation of the PTEN-PI3K-AKT pathway [52]. It was further demonstrated that *BRAF*-mutated cells acquired resistance to vemurafenib by stimulation of EGFR, thereby perpetuating cell proliferation [53•]. Based upon these findings, a triple combination of targeted agents concurrently inhibiting *BRAF*, *MEK* and EGFR is currently under investigation for treatment of *BRAF*-mutant CRC (Table 1.). Other approaches undergoing trials include use of ERK inhibitors and combinations of irinotecan, BRAF and EGFR inhibitors; BRAF, EGFR and PI3K inhibitors; BRAF, EGFR and WNT pathway inhibitors and FOLFOXIRI with bevacizumab.

### PIK3CA and PTEN

Resistance to EGFR inhibition may also be driven by the PI3K-PTEN-AKT pathway. PI3K signalling is negatively regulated by PTEN, and may be activated by *PIK3CA* mutation. This gene encodes for the catalytic subunit of PI3K and is present in 10–18 % CRC. However, activation of this pathway may also occur due to loss of PTEN expression in 30 % CRC. *KRAS* and *PIK3CA* mutation frequently coexist, with activation of parallel pathways in a single tumour. Similar to MEK inhibition, single-agent targeting of this pathway has demonstrated lack of activity due to development of resistance feedback loops [50]. Early phase clinical trials are investigating the combination of MEK inhibition with AKT, PI3K and mTOR inhibition, but results to date have been disappointing (Table 1).

Interestingly, retrospective analysis of aspirin use in patients with established CRC identified the subgroup of patients with *PIK3CA* exon 9 and 20 mutations as deriving a survival benefit [54]. Although the reason for this interaction remains poorly understood, this may provide a clinical utility of this genomic biomarker. Aspirin is being prospectively investigated in this subgroup in the UK in the FOCUS4 trial (first-line setting for metastatic disease) and in the adjuvant Add-Aspirin trial (stage II/III CRC).

### MET Gene

The mesenchymal-epithelial transition (*MET*) proto-oncogene encodes for c-MET, a receptor with tyrosine-kinase activity targeting hepatocyte growth factor (HGF). Activation of this pathway has been implicated in metastatic progression of CRC. EGFR and MET are coexpressed in CRC and *MET* activation has been implicated in resistance to the anti-EGFR mAb cetuximab [55]. c-MET is overexpressed in 50–60 %, amplified in 10 % and mutated in 5 % CRC [56, 57]. The *MET* inhibitor crizotinib was trialled as a single agent for treatment of MET-amplified CRC as part of The French National AcSé programme. However, 0/13 CRC patients with MET amplification derived clinical benefit from this agent [58]. Based upon preclinical work demonstrating synergistic activity in CRC between MEK and MET inhibitors [59], the MErCuRIC1 study is investigating the combination of the *MEK* inhibitor PD-0325901 and crizotinib in patients with *KRAS* mutant, or *KRAS* WT, MET-amplified CRC (Table 1).

### ALK/ROS1 Translocations

The *EML4-ALK* fusion gene is produced by inversion in the short arm of chromosome 2, where anaplastic large-cell lymphoma kinase (*ALK*) joins echinoderm microtubule-associated protein-like 4(*EML4*), resulting in a chimeric protein with constitutive *ALK* activity [60]. *ROS1* is an orphan receptor tyrosine kinase phylogenetically related to *ALK* [61]. These carcinogenic chromosomal translocations are found in 5 % of NSCLC, where the targeted *ALK*, *ROS1* and *MET* inhibitor crizotinib has been used with up to 74 % response rates [62, 63]. In CRC, *ALK* or *ROS1*, gene rearrangements have not been extensively studied. Several papers have detailed a unique subpopulation of between 0.8–2.5 % patients with metastatic CRC (mCRC) with either *ALK* or *ROS1* rearrangement of their tumour [64, 65]. Due to small patient numbers and inconsistent and expensive testing methods for *ROS1* translocations, developing a clinical trial of targeted therapy for this patient subset poses considerable challenges.

## Defining Molecular Subtypes in CRC: Gene Expression Profiling

Molecular subtyping of CRC has presented challenges, resulting in inconsistencies within the literature with regard to understanding of prognostic and predictive biomarkers. This may reflect the heterogeneity of CRC, possibly due to the lack of exclusivity outlined between the major pathways driving carcinogenesis and disease progression. Using gene expression profiling, the function of multiple genes can be assessed simultaneously to give a clearer picture of the complex characteristics underlying disease and reflecting genetic and epigenetic regulation. This technique may ensure a more informative result in addition to reducing the time for molecular testing and potentially has cost-saving implications for clinical practice.

### Early-Stage Disease

A number of gene expression profiling tools have been validated for clinical use to inform prognosis in stage II CRC, where the benefit for chemotherapy remains unclear [66]. Oncotype DX and ColDx were developed using formalin-fixed paraffin-embedded (FFPE) tumours, and ColoPrint used fresh frozen tissue [67–69]. Oncotype DX utilises a 12-gene signature comprising genes associated with recurrence and with clinical benefit to 5FU to stratify tumours into low-, medium- or high-risk for recurrence [67]. The ColDx (now called GeneFX colon) signature comprises a 634-gene signature which differentiates stage II tumours into low- and high-risk for recurrence. The hazard ratio for cancer-related death at 5 years was 2.21, and the test performed better than use of traditional clinicopathological variables [68]. ColoPrint uses an 18-gene signature to classify patients with stage II disease as high- or low-risk of recurrence and improved the accuracy of clinicopathological variables alone for prediction of low-risk CRC [69]. Practical difficulties with obtaining fresh frozen tissue may limit use of this assay in routine clinical practice.

### Advanced Disease

Independent investigator groups have used gene expression profiles to identify intrinsic molecular subtypes [8, 10, 70, 71]. Due to poor agreement across the resulting subtypes for several gene expression profiles, members of the Colorectal Cancer Subtyping Consortium agreed to combine their genomic datasets to generate a consensus molecular subtyping strategy (CMS) by applying unsupervised clustering techniques to 4151 samples [72••]. From this, four subsets have been established. CMS1 comprised 14 % of the patients and classified older, female patients with hypermutated MSI tumours demonstrating *BRAF* mutation and immune activation. This is consistent with the established MSI phenotype. CMS2 was the most common subset comprising 37 %. These MSS tumours displayed CIN, strong WNT/MYC pathway activation, *TP53* mutation and EGFR amplification/overexpression. These tumours were associated with a better OS and predominantly originated in the left colon. Tumours clustering as CMS3 were associated with low CIN, moderate WNT/MYC pathway activation. *KRAS* and *PIK3CA* mutation was commonly found among this subtype, as was IGFBP2 overexpression. This subset was seen in 13 % and was associated with intermediate survival. Finally, 23 % of the tumours clustered to CMS4. These were CIN/MSI heterogeneous, demonstrating mesenchymal/TGF-beta activation and NOTCH3/VEGFR2 overexpression. These patients were diagnosed at a younger age and had poorer survival outcomes. A subgroup (13 %) that remained unclassified was also identified, with no common features among these tumour tissues, and the authors suggest that these may represent a transitional phenotype or intratumoural heterogeneity. Although not a therapeutic stratifier (as it is not possible to classify individual patient tumours into these subtypes prospectively), this large dataset has facilitated greater understanding of the broad biological groups within CRC (Fig. 1b).

### Antiangiogenic Agents

There are now five antiangiogenic agents licensed for use in CRC, but empirical use of these drugs without selection contributes to underwhelming levels of efficacy. There is still no validated biomarker to predict benefit from these agents either as monotherapy or in addition to chemotherapy, despite almost two decades of investigation. A 63-gene signature has recently been shown to predict sensitivity to antiangiogenic therapy in ovarian cancer using FFPE tumour material and is currently being explored in CRC [73].

## Innovative Clinical Trial Design

With the development of novel treatment strategies for subgroups of patients with CRC, finding the optimal biomarker and ensuring appropriate targeted therapy without exhausting limited diagnostic tissue is becoming increasingly challenging. A number of trials address this issue using a basket design to stratify patients into the appropriate arm for therapy based upon the molecular characteristics of the tumour at point of testing.

The UK-based FOCUS4 study is an adaptive, biomarker-driven, multiarm, multistage trial designed to enable stratification of therapy for patients with mCRC during first-line chemotherapy (see http://www.focus4trial.org). By prospectively analysing diagnostic tumour samples for predefined biomarkers, patients with responding or stable disease following 16 weeks of first-line chemotherapy can be allocated to an appropriate treatment arm. The aim of this study is to assess response of targeted treatment strategies using novel or repurposed agents in these molecularly enriched cohorts. There is a hierarchy of biomarker-defined cohorts (Table 2) [74••].

**Table 2:** FOCUS4 trial cohorts: an example of a trial designed with treatment stratification based on molecular subtyping within colorectal cancer

Study arm	Molecular aberration	Treatment
FOCUS-A	BRAF mutation	EGFR/BRAF/MEK inhibitors vs observation
FOCUS-B	PIK3CA mutation	Aspirin vs placebo
FOCUS-C	P53 and RAS dual mutation or H3K36me3 loss	WEE1 inhibitor vs placebo
FOCUS-D	BRAF/RAS/PI3KCA WT and no PTEN loss	HER-1, 2, 3 inhibitor vs placebo
FOCUS-E	Mismatch repair deficiency or POLD1/POLE mutations	PD-L1 inhibitor vs placebo
FOCUS-F	ATM loss	ATR inhibitor vs placebo
FOCUS-N	No other cohort available	Capecitabine vs observation

MODUL is a randomised, multicentre, controlled, open-label, parallel-group study sponsored by Roche/Genentech that is investigating the efficacy and safety of biomarker-driven maintenance treatment for first-line mCRC after 16 weeks of induction of FOLFOX and bevacizumab [75]. Patients with *BRAF* mutations are randomised between fluoropyrimidine (+/−bevacizumab) maintenance and the same with addition of BRAF and EGFR inhibitors; *BRAF* WT patients are randomised between fluoropyrimidine (+/−bevacizumab) maintenance and the same with addition of a PD-L1 inhibitor.

A similar precision medicine approach is planned in the USA in second-line treatment of CRC in the ASSIGN trial [76]. Here, molecularly defined subgroups based on metastatic site biopsies will be randomised between targeted agents and chemotherapy, dependent on the availability of therapies at the time that the study opens. Current plans are to investigate novel EGFR pathway targeting agents in “all WT” CRC, BRAF inhibitors in *BRAF*-mutant CRC, targeted agents in those with ALK/ROS-1 translocations, PI3K inhibitors in those with PTEN loss, HER-2 inhibitors or TDM-1 in those with HER-2 over-expression and immune checkpoint inhibitors in those with MSI.

Screening Patients for Efficient Clinical Trial Access in advanced colorectal cancer (SPECTAcolor) is an EORTC initiative launched in 2013, comprising a network of collaborating European institutions treating patients with CRC. Tumour tissue from participants is processed for centralised, high-throughput screening for driver mutations including *KRAS*, *NRAS*, *BRAF* and *PIK3CA*, in addition to immunohistochemical staining for MSI. Next-generation sequencing will also be performed for 360 key cancer genes, with a view towards enrolment in “downstream” targeted multinational clinical trials [77]. These and similar mutational screening platforms are designed to optimise use of potentially small tissue samples in order to provide a variety of appropriately targeted additional treatment options for patients with mCRC.

## Tumour Heterogeneity

Decisions regarding therapeutic strategy are currently made using available tissue, most commonly from the primary tumour at the time of diagnosis. During progression of malignant disease, by a process of clonal evolution, molecular differences may occur between primary tumours and metastatic disease. This may render application of a biomarker to a primary tumour sample inaccurate for the purposes of targeted therapy for more advanced disease, with a lack of knowledge of the wider tumour landscape [78••]. For the clinically relevant driver genes, including *KRAS*, *NRAS*, *BRAF*, *PIK3CA* and *TP53*, concordance between tumour primary and metastatic disease site has been shown to be >90 % in CRC [79•]. There appears to be less concordance for PTEN expression by IHC, varying from 47 to 98 % between primary and metastatic site, reducing utility of this genomic marker in clinical practice [80]. Less is known about the MSI subgroup where a baseline genomically unstable tumour may become increasingly hypermutated during disease progression.

The liquid biopsy is a minimally invasive sampling technique utilising circulating cell-free (cf) DNA to reflect the dynamic nature of clonal evolution, thereby potentially eliminating inaccuracies in current molecular status of the tumour while minimising discomfort and risk to the patient [81]. Its potential utility in CRC has been demonstrated for *KRAS* and *BRAF* mutation identification during treatment [82•].

CRC has been highlighted as one of the tumour types where development of this technology has been identified as a priority. Techniques to optimise laboratory procedures for analysis and interpretation of results are currently being investigated [83]. However, validation of this exciting technology is required prior to routine clinical adoption.

## Conclusion

Although unique subtypes within CRC have been identified, critical questions remain regarding the pathogenesis and biology of these tumours, and optimal biomarkers with evidence-based therapeutic strategies remain elusive. The emergence of immunotherapy for treatment of malignancy has widened treatment options for many patients, but trials for patients with CRC are limited and are currently focused largely on those with metastatic MSI disease. The biological features of this subgroup with immune infiltration as a key feature of tumourigenesis would appear to validate this choice. However, the mechanisms underlying poor prognosis for these patients remain incompletely understood. In order to progress management of this disease, it is critical that we understand the resistance mechanisms to therapy and address these in developing the next generation of therapeutic strategies. Optimising use of EGFR inhibitors, improving treatment for those patients with *RAS* and *RAF* mutations where no proven targeted therapy exists, and for those with *RAS/RAF* WT disease resistant to EGFR inhibition, are key areas of unmet clinical need.

Enhancing our understanding of the CRC disease process may result in better use and scheduling of systemic treatment, reducing morbidity and mortality in this disease. Development of robust and validated biomarkers will be at the core of personalised therapy, and the novel trial designs currently underway will aim to rapidly isolate subpopulations for treatment while ensuring efficient use of limited tissue resources. In addition, collaboration between multiple centres will facilitate information sharing and generate more rapid trial accrual to answer these clinical questions. Although biomarkers are validated for prediction of benefit for chemotherapy in the adjuvant setting, targeted therapy has not yet demonstrated any benefit in early disease. Finally, through interrogation of the molecular and immune characteristics driving progression from adenoma to carcinoma, there is also potential for preventative measures to be explored.
